# Clustering Analysis of FDG-PET Imaging in Primary Progressive Aphasia

**DOI:** 10.3389/fnagi.2018.00230

**Published:** 2018-07-31

**Authors:** Jordi A. Matias-Guiu, Josefa Díaz-Álvarez, José Luis Ayala, José Luis Risco-Martín, Teresa Moreno-Ramos, Vanesa Pytel, Jorge Matias-Guiu, José Luis Carreras, María Nieves Cabrera-Martín

**Affiliations:** ^1^Department of Neurology, Hospital Clinico San Carlos, San Carlos Research Health Institute (IdISSC), Universidad Complutense, Madrid, Spain; ^2^Department of Computer Architecture and Communications, Centro Universitario de Mérida, Universidad de Extremadura, Mérida, Spain; ^3^Department of Computer Architecture and Automation, Universidad Complutense, Madrid, Spain; ^4^Department of Nuclear Medicine, Hospital Clinico San Carlos, San Carlos Research Health Institute (IdISSC), Universidad Complutense, Madrid, Spain

**Keywords:** primary progressive aphasia, positron emission tomography, fluorodeoxyglucose, brain metabolism, clustering analysis, frontotemporal dementia, Alzheimer's disease, unsupervised machine learning

## Abstract

**Background:** Primary progressive aphasia (PPA) is a clinical syndrome characterized by the neurodegeneration of language brain systems. Three main clinical forms (non-fluent, semantic, and logopenic PPA) have been recognized, but applicability of the classification and the capacity to predict the underlying pathology is controversial. We aimed to study FDG-PET imaging data in a large consecutive case series of patients with PPA to cluster them into different subtypes according to regional brain metabolism.

**Methods:** 122 FDG-PET imaging studies belonging to 91 PPA patients and 28 healthy controls were included. We developed a hierarchical agglomerative cluster analysis with Ward's linkage method, an unsupervised clustering algorithm. We conducted voxel-based brain mapping analysis to evaluate the patterns of hypometabolism of each identified cluster.

**Results:** Cluster analysis confirmed the three current PPA variants, but the optimal number of clusters according to Davies-Bouldin index was 6 subtypes of PPA. This classification resulted from splitting non-fluent variant into three subtypes, while logopenic PPA was split into two subtypes. Voxel-brain mapping analysis displayed different patterns of hypometabolism for each PPA group. New subtypes also showed a different clinical course and were predictive of amyloid imaging results.

**Conclusion:** Our study found that there are more than the three already recognized subtypes of PPA. These new subtypes were more predictive of clinical course and showed different neuroimaging patterns. Our results support the usefulness of FDG-PET in evaluating PPA, and the applicability of computational methods in the analysis of brain metabolism for improving the classification of neurodegenerative disorders.

## 1. Introduction

Primary progressive aphasia (PPA) is a clinical syndrome characterized by neurodegeneration of language brain systems (Mesulam et al., 2014). It may be the onset of several neurodegenerative disorders, including tauopathies, TDP-43 proteinopathies, and Alzheimer's disease (AD). Clinically, current classification distinguishes three main variants: non-fluent or agrammatic, semantic, and logopenic. Non-fluent PPA is associated with tauopathies, such as progressive supranuclear palsy, but also TDP-43 proteinopathies. The semantic variant is closely associated with TDP-43 type C pathology, and the logopenic variant may be the onset of AD in approximately 80–90 % of cases (Marshall et al., 2018). This current classification into three clinical variants has been a milestone in PPA research, because it has improved the clinical-pathological correlation (Matias-Guiu and Garcia-Ramos, 2013). However, prediction of the underlying pathology using clinical features is still incomplete, and even the usefulness of the current classification is a matter of debate.

In this regard, some studies have found a large percentage of patients not fulfilling the diagnostic criteria for a specific subtype, especially the logopenic variant (Sajjadi et al., 2012; Mesulam and Weintraub, 2014; Wicklund et al., 2014). Furthermore, other studies have suggested that apraxia of speech should be separated from agrammatic/non-fluent aphasia (Josephs et al., 2012) and have proposed alternative ways to categorize subtypes of PPA (Botha et al., 2015). Because several pathological entities have been associated with PPA (different tauopathies, AD, and three subtypes of TDP-43 proteinopathies) (Harris et al., 2013), clinical diagnosis should advance in order to improve the prediction of the underlying pathology in each individual patient. In addition, PPA patients may develop a second syndrome during the clinical course, such as atypical parkinsonian syndromes, behavioral symptoms like in behavioral variant frontotemporal dementia, dementia of Alzheimer's type, etc. (Rogalski and Mesulam, 2009; Matias-Guiu et al., 2015a). However, despite the efforts to improve cognitive and linguistic assessment of patients and their classification, the diagnosis of PPA is still challenging, and the existence of two, three or more clinical variants is controversial (Vandenberghe, 2016).

Neurodegenerative diseases are determined by a relatively specific predilection of each disease for certain brain regions and networks (Cummings, 2003; Leyton et al., 2016). ^18^F-Fluorodeoxyglucose positron emission tomography (FDG-PET) is considered a useful tool in the evaluation of patients with neurodegenerative disorders and, specifically, in PPA (Matias-Guiu et al., 2015b, 2017a). In fact, FDG-PET shows synaptic dysfunction and neurodegeneration and, hence, is a reliable biomarker, since it depicts specific brain regions impaired in each patient.

We hypothesized that performing clustering analyses of regional brain metabolism could allow an improvement in the classification of PPA patients. Thus, we aimed to study FDG-PET imaging data of a large consecutive case series of patients with PPA using unsupervised clustering algorithms in order to find out the optimal classification groups. We aimed to verify the standard three-groups classification of PPA types and, then, to discover subtype representations of the disease that could derive into different clinical course.

## 2. Methods

### 2.1. Participants

This study involved 150 FDG-PET imaging studies belonging to 91 patients with PPA (31 of them were scanned a second time during the follow-up, making a total of 122 scans) and 28 healthy controls (all of whom were scanned once). Participants were recruited consecutively in our center between November 2011 and May 2017, and they were followed-up until March 2018. Three cases with crossed aphasia (i.e., those patients with predominant right-hemisphere hypometabolism) were excluded. All patients met the current consensus criteria for PPA (Gorno-Tempini et al., 2011) and they were classified into the three clinical variants according to the diagnostic criteria (clinical and neuroimaging supported) and follow-up. Thus, clinical diagnosis of PPA variant was based on more than the initial assessment to avoid the existence of undetermined cases or the overdiagnosis of certain variants according to the current consensus criteria (Sajjadi et al., 2012; Matias-Guiu et al., 2014)

All participants underwent a detailed neurological and neuropsychological assessment, together with FDG-PET. Language assessment was performed following current recommendations for PPA (Gorno-Tempini et al., 2011), and has been described elsewhere (Matias-Guiu et al., 2015a; Matías-Guiu et al., 2017b). Amyloid imaging was available in 43 patients. PPA patients were followed every 6 months approximately, with a mean time of follow-up of 29.9 ± 17.3 months from FDG-PET imaging until the end of the follow-up or the end of the study. During follow-up, progressive supranuclear palsy, corticobasal syndrome, and amyotrophic lateral sclerosis were defined according to the diagnostic criteria for “probable”; behavioral syndrome was defined as the development of symptoms suggestive of the behavioral variant of frontotemporal dementia, such as disinhibition, dietary changes, empathy loss, etc. impacting in daily living with or without dementia; and dementia was defined as the impairment in daily-living activities due to other deficits beyond language and with no signs or symptoms suggestive of another alternative disorder (Hauw et al., 1994; Brooks et al., 2000; Armstrong et al., 2013). Healthy controls were recruited from the Department of Neurology among patients' spouses or healthy volunteers. Healthy controls were matched to the PPA group by age and gender, underwent a comprehensive neuropsychological examination to exclude cognitive deficits, and neurological diseases and suggestive symptoms were confidently ruled out. The Institutional Research Ethics Committee from our center approved the research protocol.

### 2.2. FDG-PET images acquisition and preprocessing

PET images were acquired following European guidelines (Varrone et al., 2009). Images were obtained in a Siemens Biograph True Point PET-CT scanner that integrates a 6-detector CT with a late-generation PET using lutetium oxyorthosilicate crystals. Patients fasted for at least 6 h before the scan. ^18^F-FDG (185 MBq) was administered intravenously 30 min before acquisition of images. During this period of time, patients remained at sensory rest. CT scan parameters were: kVp/effective mAs/rotation: 130/40/1; slice thickness: 3 mm; reconstruction interval: 1.5 mm; and pitch: 0.75. For PET acquisition, one bed position was obtained, and the acquisition time was 10 min.

Images were preprocessed using *Statistical Parametric Mapping 8* software (https://www.fil.ion.ucl.ac.uk/spm/). They were realigned, and normalized to the Montreal Neurological Institute standard space using a specific FDG-PET template for cognitive disorders (Della Rosa et al., 2014). Global mean normalization was performed individually. *Marsbar* software was used to perform a region of interest analysis of 116 brain areas of the Automatic Anatomical Labeling atlas belonging to the whole brain. Thus, mean uptake values were obtained for each participant in 116 regions of interest.

### 2.3. Data analysis

Clustering analysis (Hennig et al., 2015) is one of the most frequent algorithms for processing data. It is based on the idea of distance and similarity among the attributes of the samples. Recently, several types of clustering algorithms have been developed: hierarchical clustering, partitional clustering, model-based clustering, grid-based clustering and density-base clustering. Each one applies different optimization methods. In this study, we applied Agglomerative Hierarchical Clustering (HCA) as unsupervised learning algorithm (Everitt et al., 2011), particularly the *Ward Linkage* (Ward, 1963) algorithm.

HCA belongs to the special case of overlapping clustering algorithms. These iterative bottom-up classification methods create a sequence of partitions, which satisfy that $C={⋃}_{i=1}^{n}C_{i}$, where *C*_*i*_ with *i* = 1, 2, ..*n* are different partitions. The lowest level partitions are included into the highest level partitions.

The process starts clustering two closer observations and a new cluster is merged in every step. The process builds a tree structure known as *dendrogram*, and continues until all observations are clustered. The root class contains all the observations. There are several aggregation methods and, in this work, we have selected the Ward's Linkage method.

This algorithm does not guarantee finding the optimal solution, but it has demonstrated to provide a good behavior. Moreover, although the computational cost associated with Hierarchical Clustering is higher than partitional Clustering, the dendrogram obtained allows us to explore different partitions, simply by changing the cut-off level as shown in the dendrogram representation. In this way, the problem of not knowing the *k* value in advance, like for instance in the *k-means* algorithm (Mathworks, 2008), is solved. As a result, this clustering technique produces good clustering solutions (Jain and Dubes, 1988).

Ward's method works in terms of dissimilarities and it is based on the minimum variance method and the Error Sum of Squares (SSE). Ward's method estimates the proximity between clusters through their centroids. It measures the proximity between two clusters according to the increase of the SSE. Ward's method tries to minimize the sum of the squared distances for each point into the cluster, with respect to each cluster's centroid.

Dissimilarities between a cluster with *i* and *j* as components, and the rest of the objects, are computed following the *Lance-Willians dissimilarity formula*, as shown in Equation (1).

(1) $$\begin{array}{l} d\left(i⋃j,k\right)=\alpha_{i}d\left(i,k\right)+\alpha_{j}d\left(j,k\right)+\beta d\left(j,k\right)+\gamma |d\left(i,k\right)-d\left(j,k\right)| \end{array}$$

where *i* corresponds to the components in the cluster *i*, α_*i*_, α_*j*_, β, and γ are the agglomerative criterion. For the Ward's method $\alpha_{i}=\frac{\left|i|+|k\right|}{\left|i|+|j|+|k\right|}$, $\beta =\frac{\left|k\right|}{\left|i|+|j|+|k\right|}$ and γ = 0. The coordinates of the cluster center, comprising *i* and *j*, are computed as $g=\frac{|i|g_{i}+|j|g_{j}}{\left|i|+|j\right|}$, which represent a vector in the space of the set of attributes. $\frac{\left|i||j\right|}{\left|i|+|j\right|}||g_{i}-g_{j}|{|}^{2}$ computes the dissimilarity between cluster with centers *g*_*i*_ and *g*_*j*_.

The following analysis was carried out using both *Matlab* and *Weka* software. Initially, we evaluated the possibility of finding 4, 5, 6, 7, and 8 clusters in the acquired data. This process was performed in *Matlab* with the *evalclusters* function and the mentioned unsupervised learning algorithm, using the *Davies-Bouldin* criterion (Davies and Bouldin, 1979). *Evalclusters* function returned 8 as the optimal number of clusters. However, we decided to launch tests with clusters from 4 to 8, in order to analyze how patients were split into subgroups.

Davies-Bouldin (DB) index measures the similarity of clusters, and how compact a cluster is. DB depends both on the data and the algorithm. A minimum value represents a more compact cluster and therefore the clustering performed has higher homogeneity. DB is computed as $R=\frac{1}{n}\overset{n}{\underset{i=1}{\sum }}R_{i}$, where *R*_*i*_ is the maximum value for $R_{ij}=\frac{s_{i}+s_{j}}{d_{ij}}$ with *i* ≠ *j* and *d*_*ij*_ is the distance between the centers of clusters *i, j*.

After this set of experiments, the next task was to apply the Ward Linkage method according to the number of potential clusters to explore, and in order to obtain a classification of patients for this number of clusters. This process was carried out using the *Weka* software. As a result, patients were assigned to different clusters in accordance with the classification method and the data similarity.

### 2.4. Brain metabolism analysis of clusters

FDG-PET images of each obtained cluster were compared to an additional control group of 32 healthy subjects. Prior to statistical analysis, images had been spatially normalized and smoothed at 12 mm full-width at half maximum. Statistical Parametric Mapping version 8 was used for preprocessing and analysis. A two-sample T test was conducted to compare between groups, using age and gender as covariates. Statistical significance was set at *p* < 0.05 using family-wise error correction at cluster level. All voxel-based mapping analyses and statistics are shown in Table S1 (Supplementary Material).

## 3. Results

### 3.1. Description of the sample

The sample included 122 FDG-PET imaging studies from 91 patients with PPA: 46 with the non-fluent, 15 with semantic, and 61 with logopenic variants. The mean age of the PPA group was 73.48 ± 7.79, and 63 (51.6%) were women. Mean age of onset of symptoms was 70.65 ± 10.67 years. In the PPA group, at the moment of FDG-PET imaging, Mini-Mental State Examination score was 23 [interquartile range 14–27] and Addenbrooke's Cognitive Examination was 51.22 ± 23.4. Mean Functional Activities Questionnaire score was 4 [0–12].

### 3.2. Cluster analysis general results

**Figure 1:**
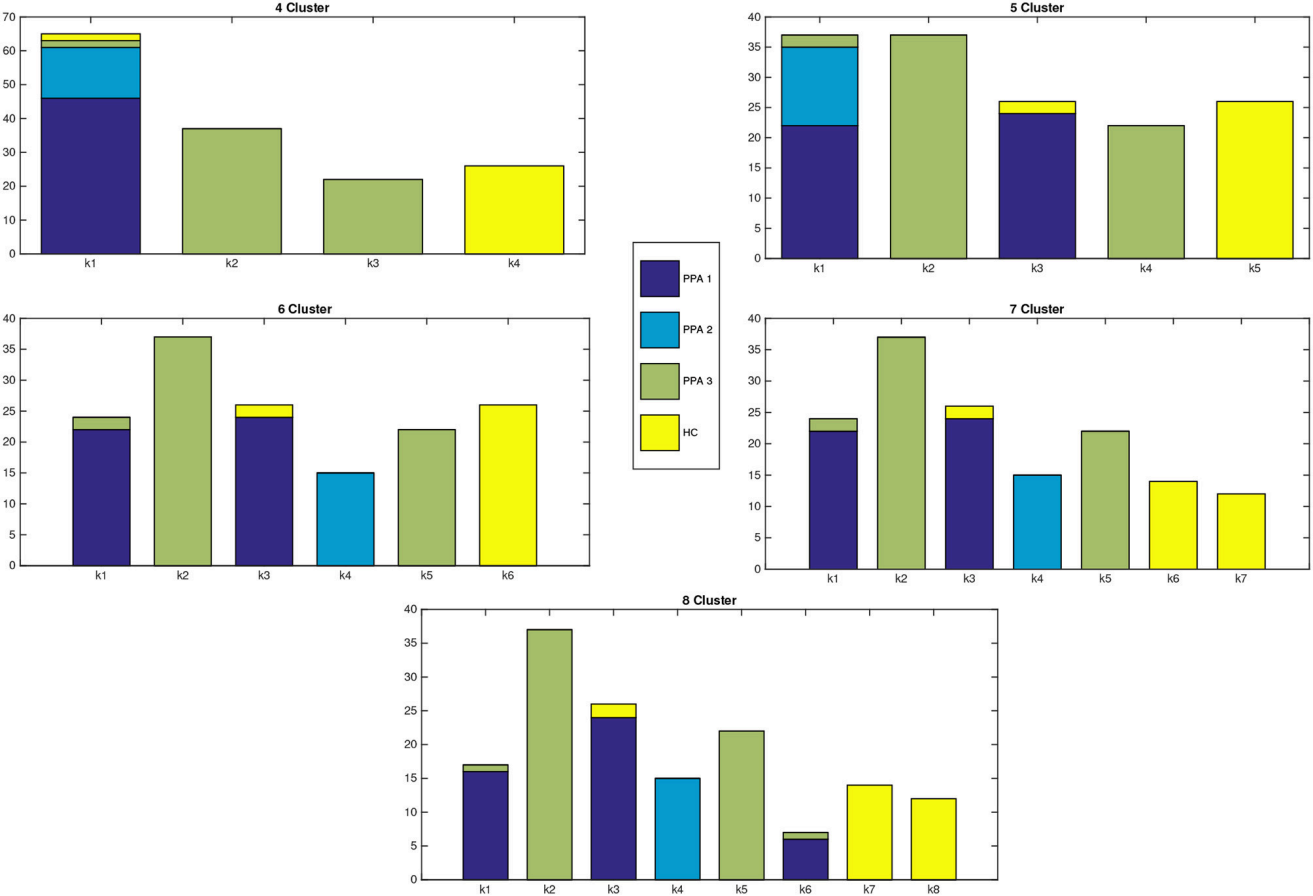
Distribution of patients within each cluster. X-axis represents the number of clusters, while in the Y-axis we show the number of patients assigned to each cluster. The different colors of the bars indicate the clinical PPA diagnosis for each patient within the cluster (1 non-fluent/agrammatic, 2 semantic, 3 logopenic) and healthy controls.

Clustering results are represented in Figure 1, where a graph for each number of clusters is drawn. For a better explanation of the results, Table 1 details the distribution of patients per cluster.

**Table 1:**
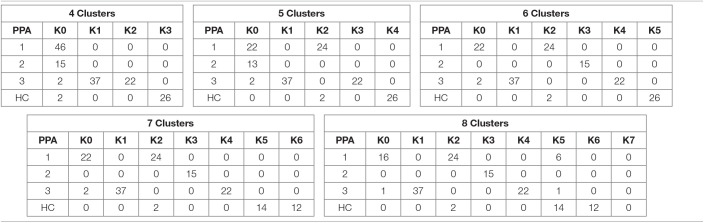
Distribution of patients per cluster and clinical PPA diagnosis for *Linkage*.

*The number of patients assigned to each cluster are shown, and their previous clinical PPA diagnosis is represented by the column labeled PPA. Values 1, 2, and 3 in the first column correspond to non-fluent/agrammatic, semantic and logopenic PPA, respectively, whereas HC represents healthy controls. Kn specifies the number of clusters, where n = 0, 1, .., N and N is a value between 4 and 8*.

**Figure 2:**
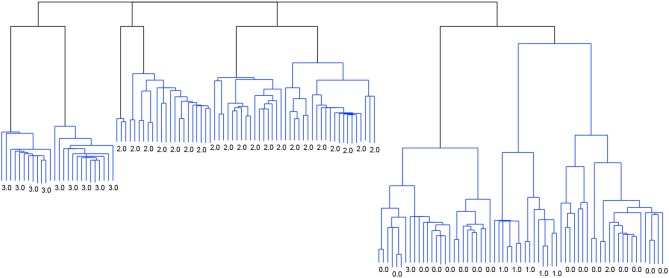
Dendrogram from the classification with *Linkage* and the criterion function *Davies-Bouldin*. 4 clusters.

**Figure 3:**
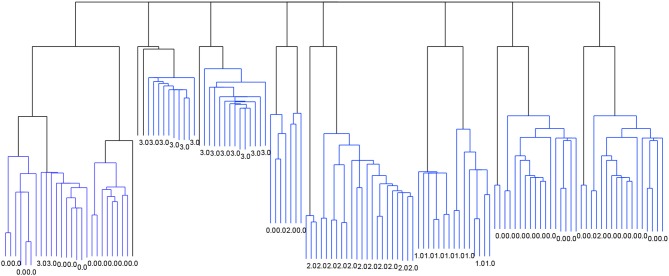
Dendrogram from the classification with *Linkage* and the criterion function *Davies-Bouldin*. 8 clusters.

Figure 2 shows the dendrogram from the 4-cluster scenario, while Figure 3 shows the dendrogram for the corresponding 8-cluster scenario.

**Figure 4:**
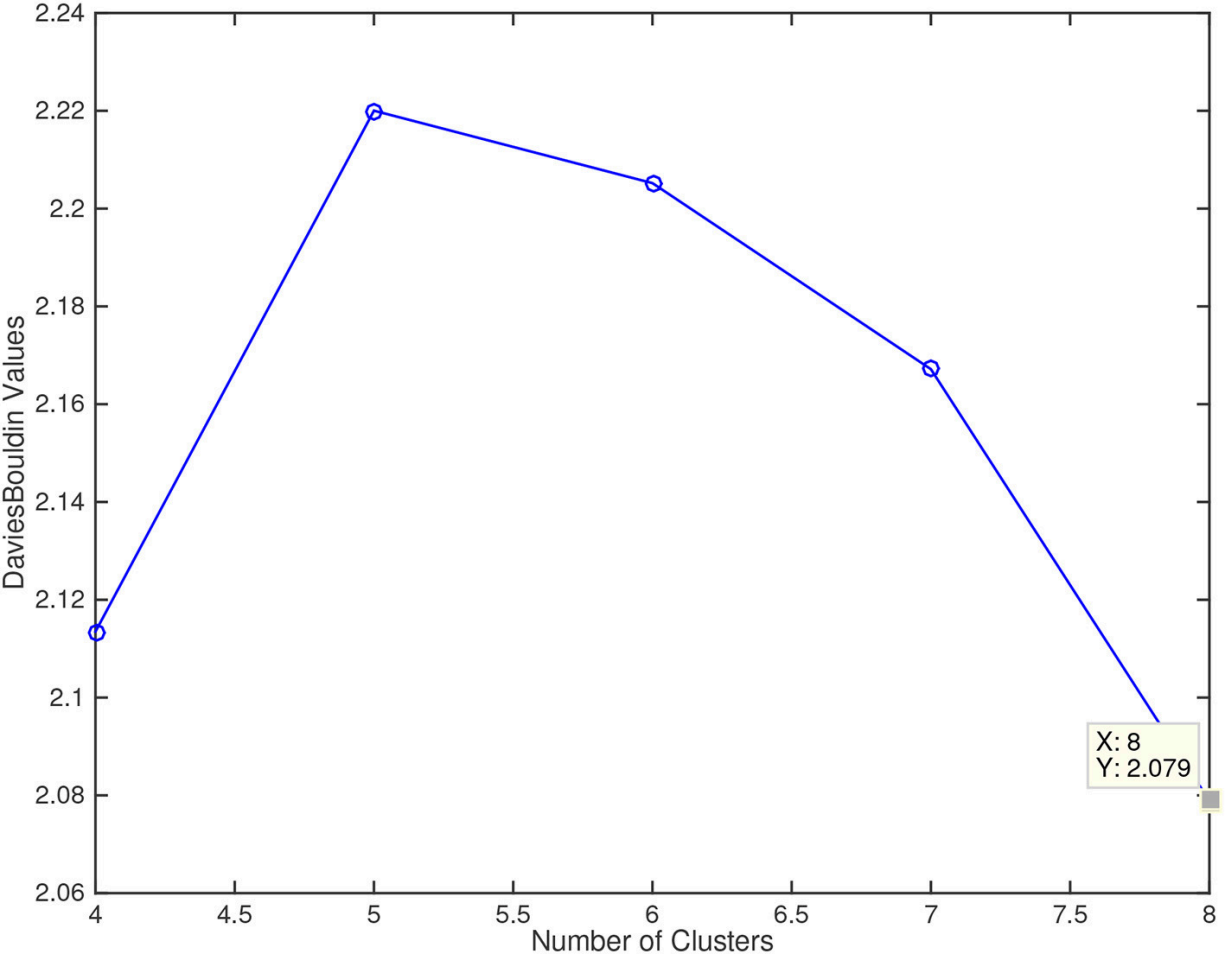
Davies Bouldin values for the number of cluster explored (4, 5, 6, 7, and 8). Lower values of this metric mean a better fitting of the sample data to the number of clusters.

The application of the *Davies-Bouldin* criterion function returned 8 as the optimal number of clusters, with an index value of 2.079, as shown in Figure 4. The figure also shows how 4 clusters could be the second best option, while 5, 6, and 7 clusters were not selected by this quality metric.

### 3.3. Clinical and neuroimaging characteristics for 4 clusters

The following groups were found when classifying for 4 clusters. The first group k0 included 65 patients, and mainly comprised patients with non-fluent and semantic PPA. The second (k1, *n* = 37) and third groups (k2, *n* = 22) were mostly patients with logopenic PPA, while k3 (*n* = 26) were healthy controls.

**Figure 5:**
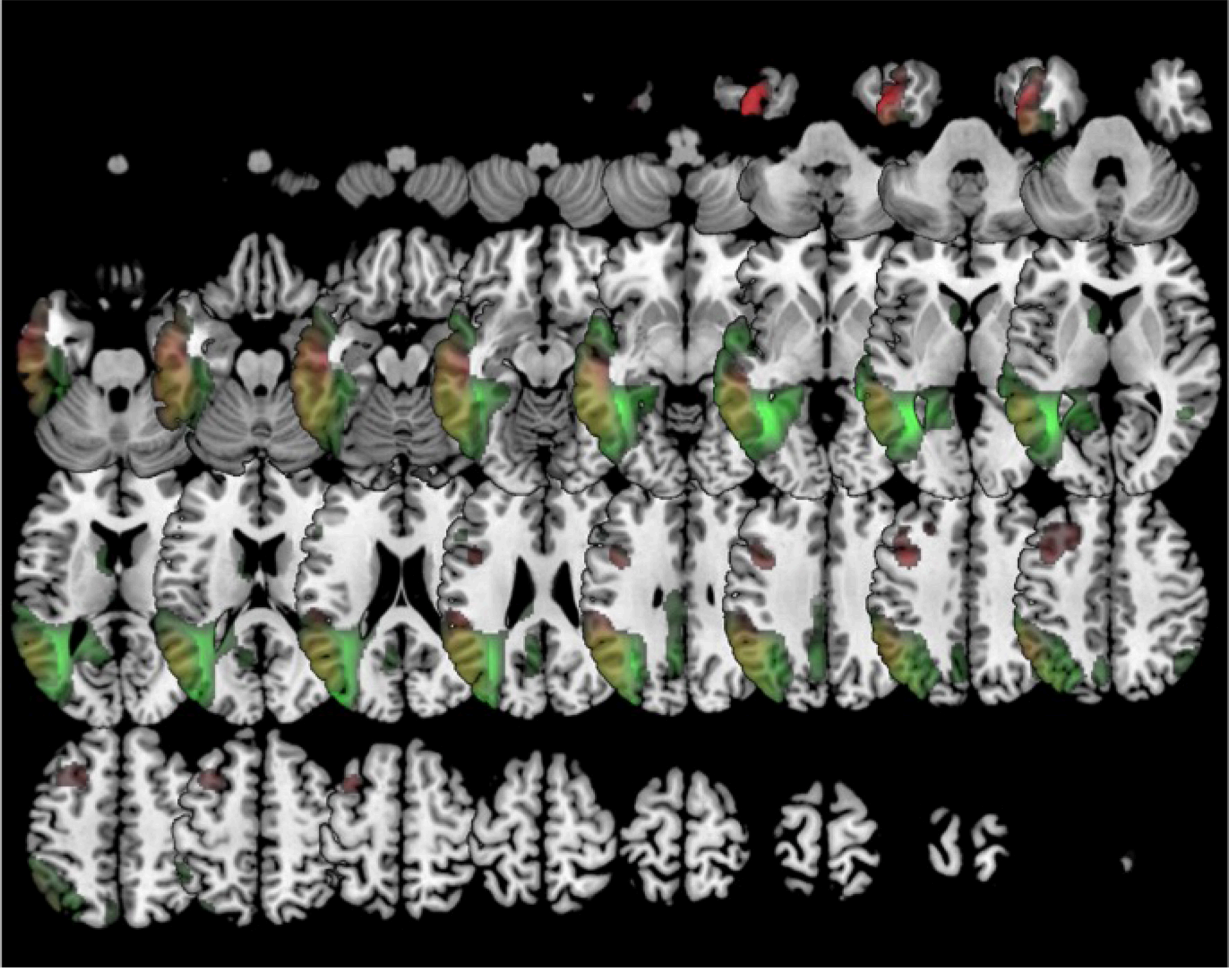
Clustering in four groups. Voxel-based brain mapping analysis showing regions with lower metabolism in the group k1 (logopenic PPA subtype 1, in red) and the group k2 (logopenic PPA subtype 2, in green) in comparison to healthy controls.

In comparison to healthy controls, k0 showed lower metabolism mainly in the left frontal lobe and the anterior temporal lobe. k1 showed hypometabolism in two main clusters: the first one involving the left supramarginal, superior, middle and inferior temporal gyri and the inferior parietal lobule; and the second one including left middle and inferior frontal gyri and precentral gyrus. k2 showed lower metabolism in a main cluster in the left hemisphere involving the middle, superior, and inferior temporal gyri, as well as fusiform, angular, and parahippocampal gyri. There was an additional cluster in the right temporal lobe and the right angular gyrus (Figure 5).

When available, amyloid biomarkers were positive in all cases classified into k1 and k2 clusters (18/18 and 15/15, respectively) and negative in 90% of cases classified into the k0 cluster (1/10 positive).

During follow-up, k0 evolved mainly to progressive supranuclear palsy (*n* = 18, 27.7 %) and behavioral symptoms (*n* = 13, 20 %). Patients within the k1 group evolved to global dementia in 26 (70.3 %). In k2, 15 (68.2 %) progressed also to dementia.

### 3.4. Clinical and neuroimaging characteristics for 5 and 6 clusters

In this analysis, groups mainly involving logopenic PPA and healthy controls remained unchanged. Conversely, the group including non-fluent and semantic variants was divided in two (in the case of 5 clusters classification) and three (in 6 clusters) groups: k0 (*n* = 24), k2 (*n* = 26), and k3 (*n* = 15). k0 and k2 were patients with non-fluent PPA, while k3 were all patients with semantic PPA.

**Figure 6:**
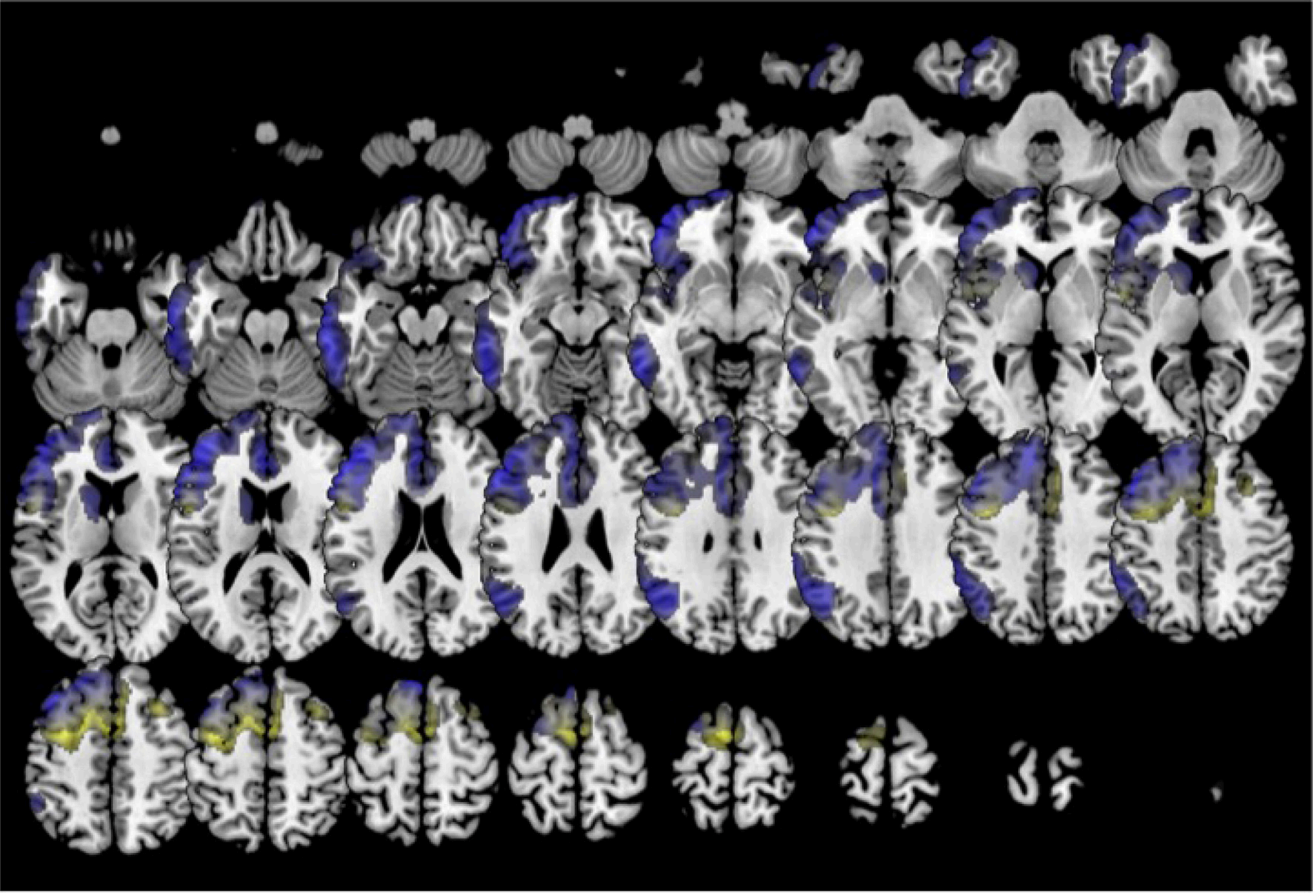
Clustering in six groups. Voxel-based brain mapping analysis showing regions with lower metabolism in the group k0 (non-fluent PPA subtype 1, in blue) and the group k2 (non-fluent PPA subtype 2, in yellow) in comparison to healthy controls.

**Figure 7:**
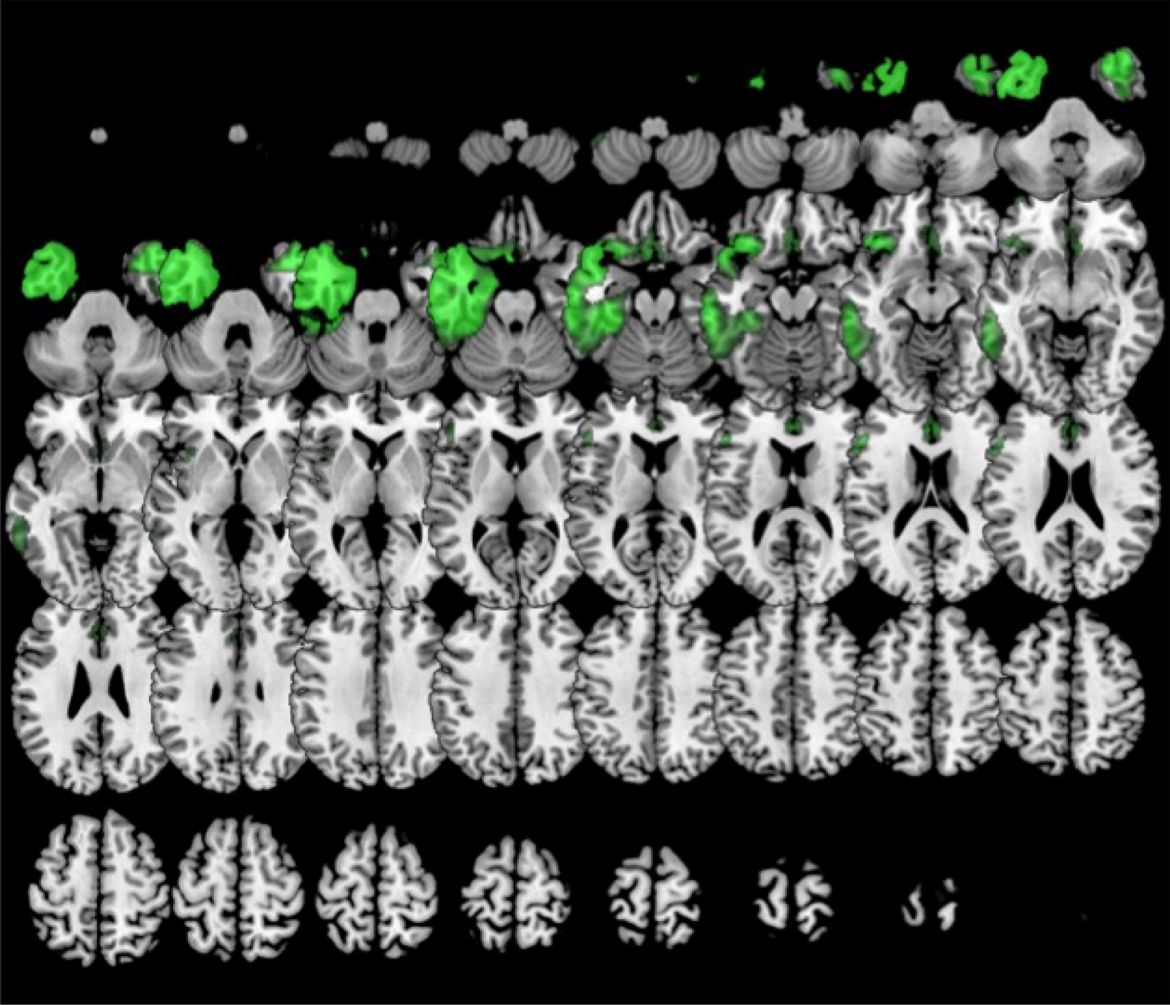
Clustering in six or eight groups. Voxel-based brain mapping analysis showing regions with lower metabolism in the group k3 (semantic PPA, in green) in comparison to healthy controls.

In comparison to healthy controls, k0 showed lower metabolism in the left frontal lobe (superior, middle, medial and inferior frontal gyru, cingulate), insula, caudate and extended also to left inferior parietal lobule and middle temporal gyrus. k2 showed lower metabolism in left frontal lobe (precentral, cingulate, middle, medial, and inferior frontal gyri) and also in the right frontal lobe (medial, middle and superior frontal, and cingulate gyri) (Figure 6). In turn, k3 showed lower metabolism in two main clusters in bilateral anterior temporal lobe (especially in the left side), and extended to some regions of the frontal lobe (Figure 7).

Amyloid imaging was negative in all cases in k0 (*n* = 3) and k3 (*n* = 6), and positive in one case in k2.

During follow-up, patients in the k0 group developed symptoms of progressive supranuclear palsy (*n* = 6, 25.0 %), global dementia (*n* = 6, 25 %), and behavioral syndrome (*n* = 4, 16.7 %). In k2, most patients evolved to progressive supranuclear palsy (*n* = 12, 46.2 %). In k3, 9 cases (60 %) developed a behavioral syndrome.

### 3.5. Clinical and neuroimaging characteristics for 7 and 8 clusters

When 7-8 clusters were considered, the former k0 group in 6 clusters was further subdivided into two subgroups (k0, *n* = 17; and k5, *n* = 7). Besides, healthy controls were also subdivided in two subgroups (k6, *n* = 14; and k7, *n* = 12). The subdivision of healthy controls in two groups probably reflects gender differences in regional brain metabolism, because 100% of cases in k6 and k7 were women and men, respectively(Hu et al., 2013).

In comparison to the healthy control group, k0 showed lower metabolism in the left frontal lobe (superior, middle and inferior frontal gyri, cingulate, insula), inferior and temporal gyri, left caudate, left inferior parietal lobule, and left rectal gyrus.

**Figure 8:**
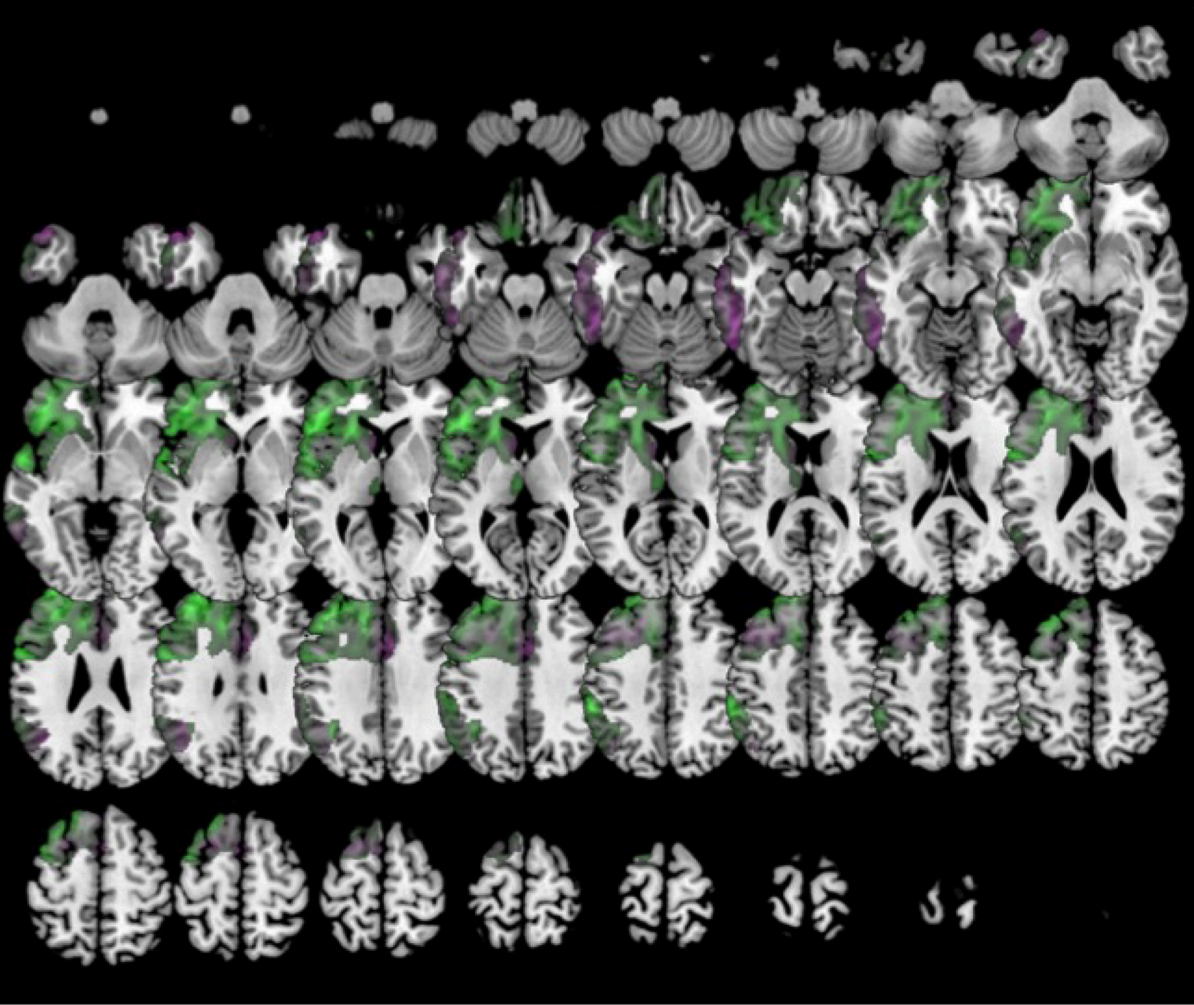
Clustering in eight groups. Voxel-based brain mapping analysis showing regions with lower metabolism in the group k0 (non-fluent PPA subtype 1A, in violet) and k5 (non-fluent PPA subtype 1B, in green) in comparison to healthy controls.

Furthermore, k5 showed lower metabolism in a large cluster involving inferior, middle and superior frontal gyri, anterior cingulate, insula and orbital gyri in the left hemisphere. k5 also showed additional regions of hypometabolism including left inferior parietal lobule and angular gyri, left inferior and middle temporal gyri, and some small clusters in right middle frontal gyrus, left thalamus, and left medial frontal gyrus (Figure 8).

**Figure 9:**
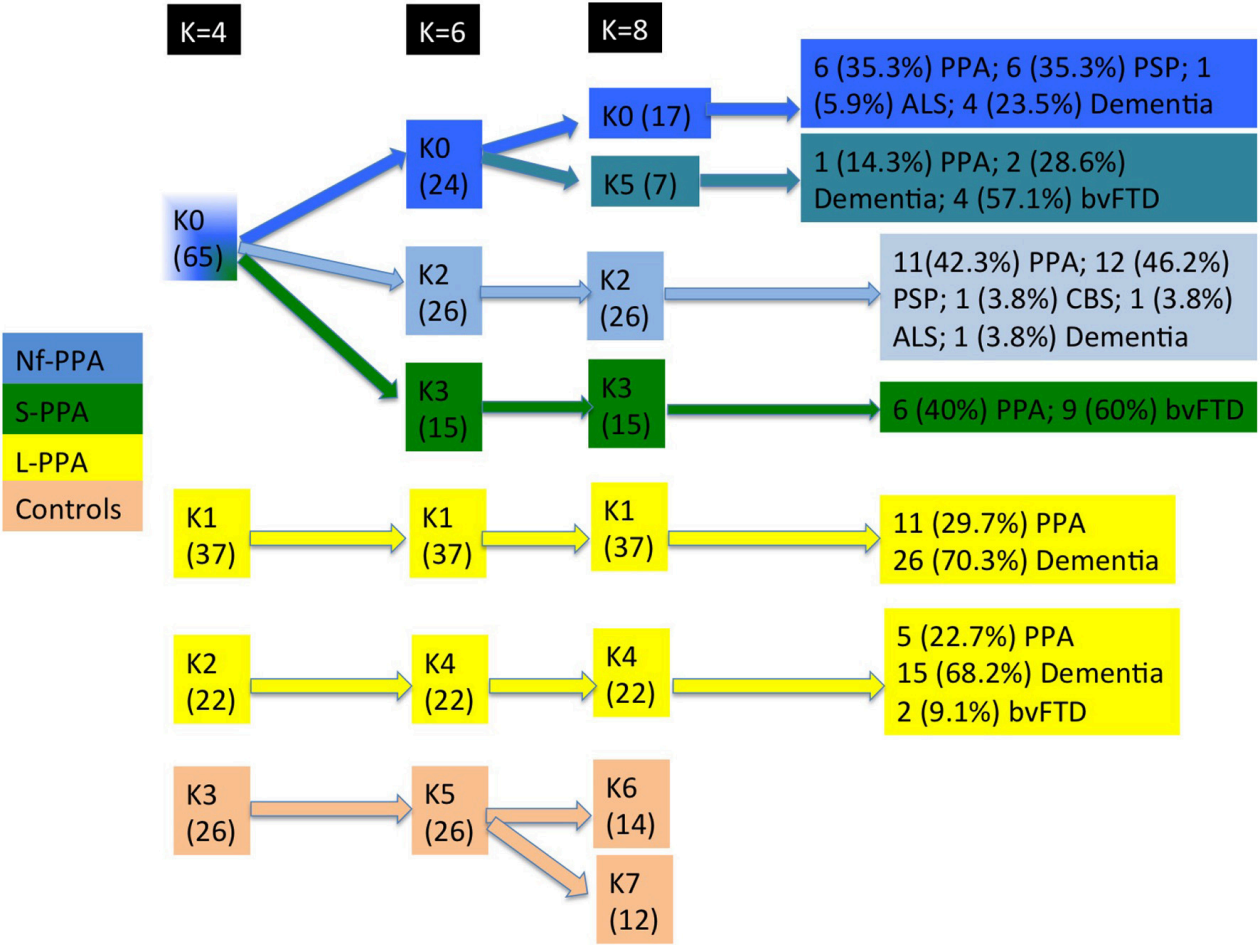
Flowchart of patient distribution within clusters, taking into account predominant clinical variants according to consensus classification and second clinical syndromes. Non-fluent PPA is shown in blue, semantic PPA in green, logopenic PPA in yellow, and healthy controls in orange. ALS, amyotrophic lateral sclerosis; bvFTD, behavioral variant frontotemporal dementia; CBS, corticobasal syndrome; PPA, primary progressive aphasia; PSP, progressive supranuclear palsy.

During follow-up, k5 evolved to dementia with (*n* = 4, 57.1 %) or without (*n* = 2, 28.6 %) prominent behavioral symptoms. k0 showed a more heterogeneous clinical course: 6 (35.3 %) developed progressive supranuclear palsy) and 4 (23.5 %) dementia with no other signs. The different clinical courses of each cluster are summarized in Figure 9.

## 4. Discussion

Our study addresses an open issue in the field of PPA regarding how patients with PPA should be classified into different subtypes. This is a very relevant question because current classification into three clinical variants aims to predict underlying pathology. Classification of PPA patients should be useful for outcome prediction, and it may be crucial in the near future when disease-modifying therapies are available. Our results confirm the current classification in non-fluent, semantic, and logopenic variants, but also suggest that current categorization may be improved.

The analysis of the distribution of patients among 4 clusters indicates that the HCA method *Linkage* distinguishes between patients associated with frontotemporal lobar degeneration (group k0), AD (groups k1 and k2) and healthy controls. This suggests, on the one hand, the capacity of FDG-PET to discriminate between frontotemporal degeneration and AD; and, on the other hand, to distinguish between PPA and healthy controls. Interestingly, patients who clinically belong to the logopenic variant were divided in two groups. Both groups showed left parieto-temporal hypometabolism. However, the first one associated left frontal hypometabolism, while the second one involved left posterior cingulate and right parieto-temporal hypometabolism. Some previous studies have suggested the existence of some subtypes within the logopenic variant, which might explain the clinical heterogeneity of this variant (Machulda et al., 2013; Leyton et al., 2015). In our series, the percentage of progression to dementia was similar in both groups.

Classification in 6 clusters divided the former group including several variants of PPA associated with frontotemporal degeneration in three subgroups: k0 (which could be called non-fluent subtype 1), k2 (non-fluent subtype 2), and k3 (which corresponds to the semantic variant). The second syndrome during the follow-up in k2 was more frequently progressive supranuclear palsy, which has been considered very specific for tauopathies 4R (Josephs et al., 2006). In contrast, non-fluent subtype 1 showed more heterogeneous progression. However, when classifying in 8 clusters (6 subtypes of PPA), k0 is subdivided in k0 (which could be named as non-fluent subtype 1A) and k5 (which could be called non-fluent subtype 1B). k5 evolved mainly to dementia with or without prominent behavioral symptoms but without parkinsonism or motor neuron disease, which might be suggestive of TDP-43 type A proteinopathy. Patterns of hypometabolism differed between groups. In the non-fluent subtype 1, hypometabolism was mainly restricted to the left hemisphere, involving left frontal lobe and also the temporal and parietal lobes. This is a neuroimaging pattern previously associated with TDP-43 proteinopathies type A (Rohrer et al., 2010; Harper et al., 2017). Conversely, non-fluent subtype 2 showed a more medial and bilateral impairment of the frontal lobe, which has been previously associated with evolution to progressive supranuclear palsy (Josephs et al., 2012; Matias-Guiu et al., 2015a). These results agree with previous studies with pathological confirmation, which have described some neuroimaging patterns more suggestive of tau than TDP-43 proteinopathies in non-fluent PPA, such as the more medial frontal and subcortical impairment, or the greater involvement of white matter than gray matter in MRI (Caso et al., 2014; Xia et al., 2017; Santos-Santos et al., 2018).

Our study suggests that FDG-PET may classify patients with PPA and, in turn, it could enable the prediction of the clinical course and possible underlying neuropathology. This is especially relevant considering some difficulties, limitations, and reduced availability of other PET tracers for amyloid and tau. In this regard, amyloid imaging may not be specific for AD, especially with aging, or it may be indicative of mixed pathology in cases associated with frontotemporal degeneration (Santos-Santos et al., 2018). In turn, tau tracers such as 18F-AV1451 showed also milder uptake in tauopathies not associated with AD or even TDP-43 proteinopathies, as has been recently outlined (Makaretz et al., 2017; Josephs et al., 2018).

According to Davies-Bouldin index, 6 subtypes of PPA (8 clusters) seem to be the a more optimized classification. We included a group of 31 patients with a second FDG-PET study during the follow-up and, in all cases, both studies were classified in the same cluster. Thus, none cluster seems to be a later stage of a previous one, and this supports the idea that each cluster represents a specific subtype of PPA. To our knowledge, this is the first study using computational analysis performed on the FDG-PET attributes in PPA, which may improve the classification of patients. Some previous studies have used data mining techniques applied to neuropsychological and language performance (Knibb et al., 2006; Wicklund et al., 2014; Maruta et al., 2015; Hoffman et al., 2017), generally confirming the non-fluent and semantic variants, but questioning the logopenic subtype. For instance, Hoffman et al. found three main clusters, the first one including the semantic variant, the second one patients with non-fluent and logopenic variants, and the third cluster with patients in more advanced stages of disease (Hoffman et al., 2017). Analysis of the topography of brain metabolism may represent a better source for computational algorithms, because it is less influenced by several factors such as culture, educational level, etc., which impact on language and cognitive assessments.

Our study has some limitations. First, although clusters are clearly defined, HCA methods, particularly *Linkage*, present as weakness that every cluster is compared only with the closest cluster. In addition, cluster analysis was based on brain metabolism in several regions of interest based on AAL atlas. Second, amyloid biomarkers were not performed in all patients, and tau imaging was not available. Further research is necessary to validate our findings in independent cohorts of patients, especially with longitudinal neuroimaging and pathological confirmation.

In conclusion, we found that unsupervised clustering analysis of FDG-PET data favored, based on the Davies-Bouldin index, the classification of PPA into six variants rather than three subtypes as currently recommended in consensus PPA criteria. These subtypes try to go beyond the current categorization in three variants, probably improving the prediction of clinical outcome. In this regard, we have identified three subtypes within non-fluent variant, two subtypes within logopenic PPA, and confirmed the semantic variant. These results also support the usefulness of FDG-PET in evaluating PPA and the possibility to improve the classification of patients with PPA using FDG-PET imaging exclusively. Furthermore, our study suggests the applicability of computational methods for clustering in the analysis of brain metabolism, which could provide new insights in neurodegenerative disorders. Future studies should evaluate clinical and language features, and longitudinal follow-up characteristics of each new subtype.

## Ethics statement

All procedures performed in studies involving human participants were in accordance with the ethical standards of the institutional research committee of the Hospital Clinico San Carlos and with the 1964 Helsinki declaration and its later amendments. Informed consent was obtained from all individual participants included in the study or their caregivers.

## Author contributions

JAM-G and JA study concept and design. JM-G and JC study supervision. TM-R and VP literature search. JAM-G, MC-M, TM-R, and VP acquisition of data. JAM-G, JD-Á, JA, and JR interpretation of data. JD-Á, JA, and JR statistical analysis of data. JAM-G, JD-Á, and JA writing the manuscript. MC-M, JM-G, and JC critical revision of the manuscript for important intellectual content.

### Conflict of interest statement

The authors declare that the research was conducted in the absence of any commercial or financial relationships that could be construed as a potential conflict of interest.
