# Supplementary material for: Clustering Analysis of FDG-PET Imaging in Primary Progressive Aphasia
Source: Front Aging Neurosci. 2018 Jul 31;10:230. doi: 10.3389/fnagi.2018.00230 (PMC6079194; doi:10.3389/fnagi.2018.00230)
Supplement: Supplementary file 1 [file Table_1.docx]

| **SUPPLEMENTARY MATERIAL. Table S1.**  Voxel-based brain mapping analysis. Regions with lower metabolism of each cluster, using a FWE corrected *p*-value <0.05. | | | | | | |
| --- | --- | --- | --- | --- | --- | --- |
| Brain region (Brodmann area) | MNI coordinates | | | T value | Z score | K (number of voxels) |
|  | x | y | z |  |  |  |
| **4 clusters. Regions with lower metabolism in k0 vs. healthy controls** | | | | | | |
| Left cingulate gyrus, inferior, middle, and superior frontal gyri, and left superior temporal gyrus (9, 10, 13, 21,24, 32, 38, 44, 46) | 0 | 20 | 30 | 9.89 | Inf | 24939 |
|  | -40 | 14 | 2 | 8.73 | 7.43 |  |
|  | -42 | 6 | 32 | 8.43 | 7.24 |  |
| Left superior temporal gyrus (39) | -48 | -60 | 30 | 7.07 | 6.30 | 1404 |
| Right superior frontal Gyrus (10) | 40 | 66 | -6 | 5.38 | 5.00 | 104 |
| Right temporal gyrus (38) | 42 | 26 | -32 | 5.10 | 4.77 | 29 |
| **4 clusters. Regions with lower metabolism in k1 vs. healthy controls** | | | | | | |
| Left Supramarginal, inferior, middle, and superior temporal gyri, inferior parietal lobule (7, 20, 21, 22, 37, 39, 40) | -52 | -54 | 32 | 11.22 | Inf | 15242 |
|  | -64 | -54 | -10 | 11.11 | Inf |  |
|  | -60 | -26 | -18 | 10.75 | Inf |  |
| Left middle and inferior frontal gyri, and precentral gyrus (6, 8, 9) | -40 | 4 | 38 | 7.12 | 6.08 | 1473 |
|  | -32 | 8 | 60 | 6.33 | 5.56 |  |
|  | -24 | 16 | 46 | 6.31 | 5.54 |  |
| Right middle temporal gyrus (39) | 50 | -62 | 30 | 5.09 | 4.65 | 37 |
| Left precentral gyrus (44) | -52 | 10 | 12 | 4.93 | 4.52 | 2 |
| **4 clusters. Regions with lower metabolism in k2 vs. healthy controls** | | | | | | |
| Left middle, superior and inferior temporal gyri, fusiform, angular, and parahippocampal gyri (19, 20, 21, 22, 37, 39) | -42 | -54 | -2 | 14.81 | Inf | 24431 |
|  | -64 | -50 | -6 | 14.54 | Inf |  |
|  | -40 | -56 | 34 | 12.81 | Inf |  |
| Right middle and superior temporal gyri, angular gyrus (20, 39) | 48 | -66 | 28 | 8.11 | 6.43 | 1318 |
|  | 48 | -52 | 12 | 7.57 | 6.13 |  |
|  | 52 | -42 | -14 | 6.31 | 5.37 |  |
| Left caudate | -10 | 6 | 8 | 5.73 | 4.99 | 128 |
| Left precuneus (7) | -10 | -88 | 52 | 5.70 | 4.97 | 22 |
| Left posterior cingulate (31) | -6 | -32 | 32 | 5.35 | 4.73 | 22 |
| Left precentral gyrus (9) | -40 | 6 | 34 | 5.34 | 4.71 | 12 |
| **6 clusters. Regions with lower metabolism in k0 vs. healthy controls** | | | | | | |
| Left superior, middle, medial and inferior frontal gyri, cingulate, insula and caudate (6, 8, 13, 32, 38, 45) | -8 | 30 | 56 | 10.05 | 7.43 | 28865 |
|  | -40 | 20 | 46 | 9.52 | 7.18 |  |
|  | -4 | 18 | 32 | 9.55 | 7.20 |  |
| Left middle temporal gyrus, inferior parietal lobule and supramarginal gyrus (39, 40) | -44 | -60 | 30 | 7.74 | 6.27 | 2704 |
|  | -64 | -36 | 40 | 7.72 | 6.25 |  |
|  | -56 | -52 | 32 | 7.46 | 6.11 |  |
| Right Superior Frontal Gyrus (10) | 40 | 66 | -4 | 6.41 | 5.46 | 265 |
| Left rectal gyrus (11) | -8 | 18 | -28 | 6.06 | 5.23 | 227 |
|  | -10 | 36 | -26 | 5.23 | 4.66 |  |
| Left thalamus | -4 | -18 | 8 | 5.08 | 4.55 | 3 |
| **6 clusters. Regions with lower metabolism in k2 vs. healthy controls** | | | | | | |
| Left precentral, middle frontal, medial frontal, inferior frontal cingulate gyri; right medial frontal and cingulate gyri (6, 8, 9, 32, 45) | -42 | 4 | 34 | 10.23 | 7.57 | 11435 |
|  | -52 | 6 | 16 | 9.06 | 7.01 |  |
|  | -4 | 16 | 46 | 8.88 | 6.92 |  |
| Right middle and superior frontal gyrus (6) | 30 | 16 | 50 | 6.86 | 5.78 | 572 |
|  | 22 | 16 | 62 | 5.76 | 5.05 |  |
| Left caudate | -14 | 10 | 4 | 6.45 | 5.52 | 175 |
| Left superior temporal gyrus (38) | -46 | 20 | -36 | 5.37 | 4.78 | 7 |
| Left superior frontal gyrus (10) | -14 | 66 | 16 | 5.05 | 4.54 | 1 |
| **6 clusters. Regions with lower metabolism in k3 vs. healthy controls** | | | | | | |
| Left inferior temporal, superior temporal, uncus, parahippocampal gyri, left orbital and rectal gyri (20, 36, 38, 47) | -36 | -2 | -46 | 20.23 | Inf | 14019 |
|  | -44 | 16 | -32 | 18.88 | Inf |  |
|  | -38 | -28 | -26 | 12.03 | Inf |  |
| Right superior Temporal and uncus (20, 38) | 34 | 16 | -40 | 11.23 | 7.59 | 2011 |
|  | 38 | -6 | -44 | 8.00 | 6.20 |  |
| Left anterior cingulate (24) | -2 | 30 | 20 | 7.60 | 6.00 | 525 |
| Left inferior frontal gyrus (45) | -54 | 22 | 18 | 7.41 | 5.89 | 360 |
|  | -50 | 30 | 8 | 6.17 | 5.17 |  |
| Left middle and superior frontal gyrus (10) | -38 | 62 | -8 | 6.60 | 5.44 | 310 |
|  | -26 | 68 | -10 | 6.57 | 5.42 |  |
| Left superior frontal gyrus (8) | -8 | 46 | 40 | 5.48 | 4.73 | 18 |
| Left cingulate (24) | -2 | 8 | 36 | 5.25 | 4.58 | 2 |
| **8 clusters. Regions with lower metabolism in k0 vs. healthy controls** | | | | | | |
| Left Superior, middle and inferior frontal gyri, cingulate gyrus, insula, inferior and superior temporal gyri (6, 8, 13, 20, 32, 38, 45) | -8 | 30 | 58 | 9.79 | 7.10 | 18907 |
|  | -52 | 26 | 20 | 9.66 | 7.04 |  |
|  | -40 | 22 | 46 | 9.54 | 6.99 |  |
| Left Inferior Parietal Lobule and superior temporal gyrus (39, 40) | -44 | -60 | 30 | 7.38 | 5.92 | 1490 |
|  | -64 | -34 | 46 | 6.82 | 5.60 |  |
|  | -54 | -48 | 42 | 6.44 | 5.38 |  |
| Left caudate | -12 | 12 | 4 | 6.71 | 5.54 | 390 |
| Right Superior Temporal Gyrus (38) | 42 | 26 | -34 | 5.65 | 4.87 | 18 |
| Left rectal gyrus (11) | -8 | 16 | -28 | 5.62 | 4.85 | 19 |
| Left Rectal superior frontal gyrus (10) | 28 | 72 | -8 | 5.60 | 4.84 | 54 |
|  | 38 | 68 | -4 | 5.41 | 4.71 |  |
| **8 clusters. Regions with lower metabolism in k5 vs. healthy controls** | | | | | | |
| Left inferior frontal, middle frontal, superior frontal gyri, anterior cingulate, insula and left orbital gyrus (10, 13, 32, 45, 47) | -52 | 24 | 18 | 14.46 | Inf | 24815 |
|  | -48 | 48 | -4 | 12.71 | 7.66 |  |
|  | -54 | 6 | 16 | 12.18 | 7.51 |  |
| Left Inferior Parietal Lobule and angular gyrus (39, 40) | -58 | -38 | 42 | 9.91 | 6.75 | 2054 |
|  | -40 | -54 | 36 | 8.18 | 6.04 |  |
|  | -56 | -54 | 38 | 8.08 | 5.99 |  |
| Right Middle Frontal Gyrus (10) | 40 | 64 | -2 | 8.05 | 5.98 | 160 |
| Left inferior and middle temporal gyri (37) | -64 | -48 | -8 | 7.83 | 5.88 | 1875 |
|  | -64 | -36 | -16 | 7.55 | 5.74 |  |
|  | -56 | 0 | -38 | 7.48 | 5.71 |  |
| Left thalamus | -6 | -18 | 8 | 7.71 | 5.82 | 139 |
| Left medial frontal gyrus (10) | -8 | 46 | -8 | 6.20 | 5.04 | 66 |
